# Development of surface plasmon resonance-based sensor for detection of silver nanoparticles in food and the environment

**DOI:** 10.1007/s00216-012-5920-z

**Published:** 2012-03-27

**Authors:** Sabina Rebe Raz, Maria Leontaridou, Maria G. E. G. Bremer, Ruud Peters, Stefan Weigel

**Affiliations:** RIKILT—Wageningen University and Research Centre, P.O. Box 230, 6700 AE Wageningen, The Netherlands

**Keywords:** Silver nanoparticles, Metallothionein, Food, Environment, Surface plasmon resonance

## Abstract

Silver nanoparticles are recognized as effective antimicrobial agents and have been implemented in various consumer products including washing machines, refrigerators, clothing, medical devices, and food packaging. Alongside the silver nanoparticles benefits, their novel properties have raised concerns about possible adverse effects on biological systems. To protect consumer’s health and the environment, efficient monitoring of silver nanoparticles needs to be established. Here, we present the development of human metallothionein (MT) based surface plasmon resonance (SPR) sensor for rapid detection of nanosilver. Incorporation of human metallothionein 1A to the sensor surface enables screening for potentially biologically active silver nanoparticles at parts per billion sensitivity. Other protein ligands were also tested for binding capacity of the nanosilver and were found to be inferior to the metallothionein. The biosensor has been characterized in terms of selectivity and sensitivity towards different types of silver nanoparticles and applied in measurements of real-life samples—such as fresh vegetables and river water. Our findings suggest that human MT1-based SPR sensor has the potential to be utilized as a routine screening method for silver nanoparticles, that can provide rapid and automated analysis dedicated to environmental and food safety monitoring.

**Electronic supplementary material:**

The online version of this article (doi:10.1007/s00216-012-5920-z) contains supplementary material, which is available to authorized users.

**Figure:**
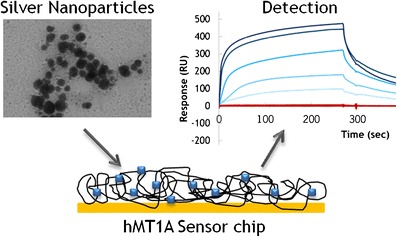
Surface plasmon resonance biosensor for rapid detection of silver nanoparticles was developed. Human metallothionein 1A (hMT1A) was immobilized on the sensor chip surface and showed dose dependent binding to the silver nanoparticles with part per billion sensitivity.

## Introduction

Recent advances in nanotechnology have introduced novel nanomaterials (NM) into our environment. Due to their small size, the NM exhibit different physicochemical properties compared to their respective bulk material. Their benefits led to an increasing amount of applications in many sectors including electronics, clothing, medicine, cosmetics and food [1]. Alongside titanium oxide and silica, nanosilver is one of the most perspective and widespread NM [2]. Silver-based compounds are recognized as effective antimicrobial agents and have been implemented in various medical applications [3, 4]. Also, silver nanoparticles (AgNPs) were found to inhibit bacterial growth. It was suggested that AgNPs can be applied to prevent deleterious infections in a cost-effective manner, enabling the development of new types of bactericidal devices [5–8]. Indeed AgNPs are currently used in several consumer products: washing machines, refrigerators and clothing [1]. In the food industry, the AgNPs are used to develop new packaging materials with improved antimicrobial properties [8, 9]. Alongside the AgNPs benefits, their novel properties have raised concerns about possible adverse effects on biological systems [10, 11]. Recently, Wenjuan et al. reported nanosilver interference with DNA replication fidelity and binding to DNA [12]. AgNPs were also found to halt the reproduction activity of benign species of bacteria, which are used for wastewater treatment and to induce cellular and DNA damage in aquatic organisms [13, 14]. Currently, there is no legislation in place for silver nanoparticles, nonetheless the European Scientific Committee makes a series of recommendations on actions should be taken to develop methods to detect and measure engineered nanomaterials, such as AgNPs, in food and feed [15–17].

A range of analytical techniques is available for the detection and characterization of pure AgNPs suspensions, including electron and atomic force microscopy, size exclusion chromatography, filtration techniques and light scattering methods. All the existing methods suffer from high costs, extensive sample preparation and require long analysis times or are not specific (e.g., light scattering) [18–20]. Furthermore, for complex matrices such as environmental and food samples the methods are very limited[1]. Besides instrumental analytical methods, which are required for appropriate risk assessment, fast screening assays are needed for efficient environmental and food safety monitoring. The screening assays enable informative decision making on prioritization of further testing in more depth [21]. To the best of our knowledge, so far, there has been no such assay developed for detection of the silver in its nanoform, only for silver ions [22, 23].

In the last decade optical biosensors, based on surface plasmon resonance (SPR) phenomenon, became well acknowledged screening tools, that provide a real-time and automated analysis with relatively high capacity [24]. Incorporation of the biological recognition elements to the sensor surface allows detection of potentially biologically active compounds. For instance, the detection of bioavailable heavy metals can be achieved through the use of metal binding proteins such as metallothioneins (MTs). MTs belong to a family of low molecular weight proteins (6–7 kDa for mammalian) capable of binding metal ions through the intrinsic 20 cysteinyl groups [25]. They participate in multiple biological processes including essential metals homeostasis, detoxification of toxic metals and cell protection against oxidative damage [26]. The use of MTs in assays for environmental monitoring enables detection of metal ions such as cadmium, zinc, nickel, and mercury [27, 28]. For silver ions detection, only an electrochemical sensor, based on rabbit liver MT, has been reported [22, 23]. Currently, there is no screening method available for the detection of the silver in its nanoform, the AgNPs. Here, we describe the development of human metallothionein 1A (hMT1A)-based sensor for the detection of AgNPs in the environmental and food samples.

## Materials and methods

### Chemicals and materials

Silver wire, diameter 2 mm and 99.99 % purity, was purchased from Sigma-Aldrich (St. Louis, MO, USA). Suspensions of citrate stabilized BioPure AgNPs (20, 30, 60, and 110 nm diameter) were purchased from NanoComposix (San Diego, USA). Human recombinant metallothionein 1A was purchased from Abnova Corporation (Taipei, Taiwan). One hundred-nanometer-thick carboxymethylated dextran (CM5) sensor chips, amine coupling kit (containing 0.1 M *N*-hydroxysuccinimide (NHS), 0.4 M *N*-ethyl-*N*-(3-dimethylaminopropyl) carbodiimide hydrochloride (EDC)) were purchased from GE Healthcare (Uppsala, Sweden). Anti-bovine IgG was purchased from Abcam (Cambridge, UK). Cadmium nitrate, zinc nitrate, nickel nitrate, manganese nitrate, calcium nitrate, magnesium nitrate and iron nitrate ICP standards were purchased from Merck (Darmstadt, Germany). The rest of the chemicals were purchased from Sigma-Aldrich (Zwijndrecht, The Netherlands).

### AgNPs preparation

AgNPs were produced electrochemically using a method comparable to the one described by Khaydarov et al. [29]. The electrodes were prepared from pure silver wires, 2 mm diameter and 40 mm long. The electrodes were placed 30 mm apart and immersed in a beaker glass containing 150 ml of distilled water. Electrolysis was performed at a constant current of 2 mA. At regular intervals, the electrodes were removed from the water and the debris was removed from the electrodes with a clean piece of cloth. At the same time, the water in the beaker glass was heated in a microwave oven to almost 100 °C and this cycle was repeated when the temperature of the water had decreased to about 40 °C. After a few cycles, the distilled water obtains a bright yellow-green color indicating the presence of AgNPs. The AgNPs suspension was stored under ambient conditions in dark-brown glass containers.

### AgNPs characterization

Transmission electron microscopy (TEM) was used to observe the size, shape and morphology of AgNPs using a JEOL JEM1011 transmission electron microscope (Tokyo, Japan). The images were analyzed with ImageJ software (ver.1.44). AgNPs were suspended in pure water at a concentration of 5 μg ml^−1^ and were let to dry on Formvar Carbon Film 400-Cu grids before the TEM imaging. The size of AgNPs was also evaluated by dynamic light scattering (DLS) measurements using ALV–L laser goniometer. The size of AgNPs was measured in pure water and in 20 mM HEPES pH 7.4 at a concentration of 1 μg ml^−1^. The concentration of the electrochemically synthesized AgNPs was determined by single particle inductively coupled plasma mass spectrometry (sp–ICPMS) using commercially obtained 60 nm AgNPs for calibration. Zeta potential of the AgNPs was determined using a Zetasizer (Malvern Instruments Ltd., UK) at a concentration of 1 μg ml^−1^ 20 mM HEPES pH 7.4, pure water and regeneration solution.

### Preparation of MT1-coated sensor chip

MT1 was immobilized on a CM5 sensor chip using a common amine coupling chemistry in Biacore 3000 [30]. Briefly, the sensor chip surface was activated with 1:4 mixture of NHS and EDC for 7 min. MT1 was dissolved in 10 mM acetate buffer pH 4 to a concentration of 10 μg/mL and injected at 20 μl/min to the activated flow channel for 10 min. The remaining reactive groups on the sensor surface were blocked with 1 M ethanolamine pH 8.5. For reference, a blank flow channel (FC) was prepared by EDC/NHS activation and deactivation with ethanolamine without introducing the protein to the surface. For comparison, flow channels with ovalbumin and IgG were prepared in the same way.

### Surface plasmon resonance measurements

All the SPR measurements were performed in the Biacore 3000 using 20 mM HEPES pH 7.4 as the running buffer. A temperature of 37 °C was found to be optimal for the MT1 interaction and was maintained constant during the measurements. Six dilutions of AgNPs in the running buffer were prepared at concentrations ranging from 0.2 μg/L to 8.7 mg/L and injected to the MT1-coated flow channel at 70 μl/min for 4.5 min. The surface was regenerated after each sample by a duplicate 30-s-long injection of 100 mM NaOH. Raw sensorgrams were double referenced, first to the blank injections of buffer and then to the blank flow channel. The binding responses were taken at the maximum of the interaction phase, 10 s prior to the end of the AgNPs injection. The responses were plotted against the AgNPs concentration and fitted with a sigmoidal curve according to the following equation: $Y = {\text{Bottom}} + {{{\left( {{\text{Top}} - {\text{Bottom}}} \right)}} \left/ {{\left( {1 + {{10}^{ \wedge }}\left( {\left( {{\text{LogEC}}50 - X} \right)} \right)} \right)}} \right.}$, where *Y* is the SPR signal, *X* is the AgNPs concentration, Top and Bottom are plateaus in the units of the *Y* axis, EC50 is the concentration that gives a response half way between Bottom and Top. Then, the concentration of AgNPs that gives a response half way between Bottom and Top (EC_50_) was interpolated. For measurements in food and water extracts, blank injections of the food or water matrix at the corresponding dilutions were used for referencing. The sensor chips were continuously monitored for their stability by comparing the calibration curves obtained with ES AgNPs. When deviation of more than 20 % was observed the sensor chip was discarded.

### Food and water extracts preparation

Fresh tomato and cucumber were homogenized separately using a food processor. Five-, 10-, and 20-fold dilutions for cucumber and 5-, 20-, and 100-fold dilutions for tomato were prepared by diluting 1.23, 4.25, 4.9 mg of cucumber and 0.22, 1.23, and 4.9 mg of tomato to a final volume of 25 ml in 20 mM HEPES pH 7.4. The extracts were then centrifuged for 10 min at 3,220×*g* and the supernatants were spiked with 100 μg/mL electrochemically synthesized AgNPs. River water was collected from the Lower Rhine (Nederrijn), in Wageningen the Netherlands. The sample container was first washed with river water three times and then the samples were collected by submerging the container in the water and closing while it was still submerged. The collected river water was filtered using a 0.22-μm polystyrene filter, to prevent microbial growth. Prior to SPR measurements the water was buffered to 20 mM HEPES pH 7.4, diluted 5-, 20-, and 100-fold and spiked with 100 mg/L electrochemically synthesized AgNPs. All spiked extracts were filtered with 5 μm syringe filter prior to injection to prevent flow channels blockage by large aggregates, if formed.

## Results and discussion

### hMT1A-sensor assay set-up

**Fig. 1:**
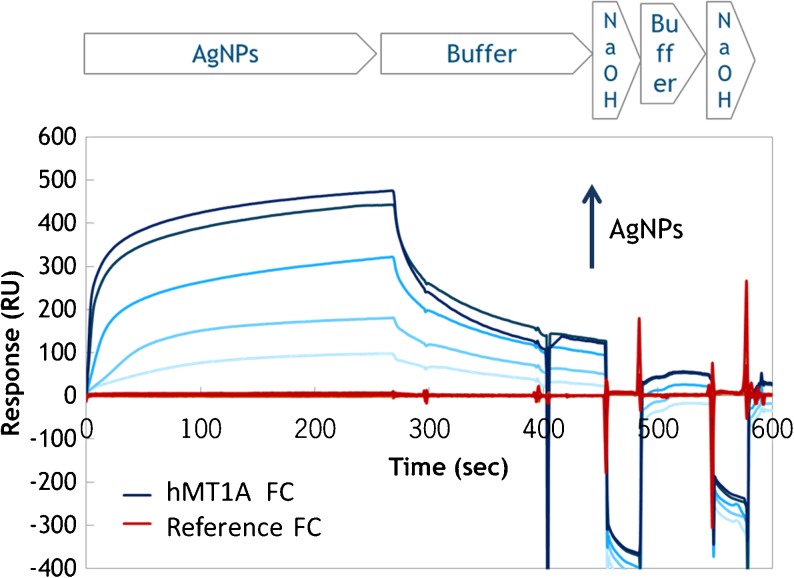
Interaction of human metallothionein 1 A (hMT1A) with increasing concentrations of 20 nm NC AgNPs (0–8.7 mg/L) as monitored using surface plasmon resonance. Sensorgrams obtained on hMT1A-coated and on reference flow channels are shown in *blue* and *red lines* respectively. Each measurement cycle included sample injection, dissociation in buffer, regeneration with NaOH, buffer wash and a second regeneration with NaOH

The aims of this study were to set-up and evaluate a screening assay for AgNPs using hMT1A in combination with SPR-based label-free detection. Even though the interaction of hMT1A with metal ions has been extensively studied, the binding to metals in their nanoform has not been reported yet. First step towards functional SPR-based biosensor is the sensor chip surface modification with the biological recognition element, the ligand. When assays for concentration measurements are designed in SPR-based biosensors, a maximal load of the ligand on the surface is usually required. Additionally, the sensor chip should be of multiple usages and has to withstand multiple cycles of regeneration without major losses of the ligands activity. Therefore, we chose covalent immobilization the hMT1A via its primary amine groups on the carboxymethylated dextran sensor chip using commonly applied EDC/NHS chemistry [30]. The immobilization level reached 7611 RU, indicating roughly 8 ng/mm^2^ hMT1A surface density. The activity of the immobilized hMT1A was assayed with 20 nm BioPure NanoComposix (NC) AgNPs. The AgNPs were diluted in 20 mM HEPES buffer pH 7.4 (running buffer) and injected to the hMT1A-coated FC. AgNPs showed dose-dependent binding to the immobilized hMT1A (Fig. 1). No binding was observed on the reference FC. The interaction conditions were adjusted by tuning the temperature, flow rate and regeneration solution. Maximal binding responses and sufficient regeneration of the sensor surface with maintained hMT1A activity were achieved using 70 μl/min flow rate at 37 °C with 100 mM NaOH as the regeneration solution. These conditions are largely in agreement to previously reported MT-based sensors for silver ions detection with exception of the regeneration solution. In most reported cases, the interaction of MT with metal ions was disrupted using either a metal chelator (e.g., EDTA), high salt solution or an acidic buffer [27, 28, 31]. In our case, these regeneration solutions were found to be ineffective. Most likely, this is due to the fact that each AgNP is capable of binding to several hMT1A molecules simultaneously and by so cross-linking the hMT1A-modified carboxymethylated dextran hydrogel, consequently requiring more stringent conditions for surface regeneration, e.g. 100 mM NaOH. The association phase with the AgNPs lasted 4.5 min, followed by dissociation in the running buffer and double injection of regeneration solution, adding up to a 10-min-long measurement cycle. The sensor chip was stable for at least 200 measurement cycles, over a period of 4 months.

### Comparison of protein ligands for AgNPs

**Fig. 2:**
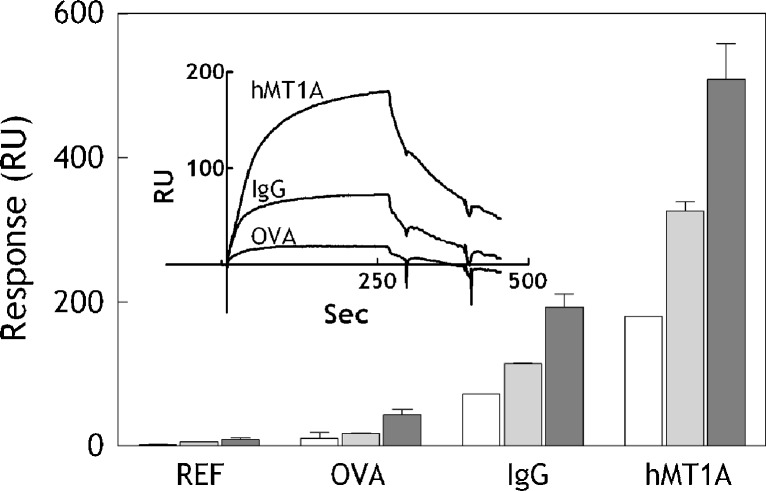
Binding of AgNPs to different protein ligands. Ovalbumin (OVA), immunoglobulin G (IgG) and human metallothionein 1 A (hMT1A) were covalently immobilized on separate flow channels at equal levels of 7,000 RU. The reference channel (REF) was activated and de-activated without the presence of protein. 20 nm NC AgNPs were injected at three different concentrations (0.07, 0.4, and 8.7 mg/L) and the maximal binding responses were compared. *Error bars* show standard deviations between three independent experiments. Inset shows raw sensorgrams obtained on hMT1A, IgG and OVA flow channels during injection of 0.07 mg/L 20 nm NC AgNPs

The binding of MT’s to heavy metals is mediated through thiols [25]. Thus, in order to compare hMT1A with other protein ligands for AgNPs detection, the remaining FC’s of the sensor chip were modified with ovalbumin (OVA) containing one disulfide bond and immunoglobulin G (IgG) containing multiple disulfide bonds. Both OVA and IgG were immobilized at the same level as the hMT1A to enable direct comparison. NC AgNPs were injected at different concentrations (0.07, 0.4, and 8.7 mg/L) and the maximal binding responses obtained on all four FCs were compared (Fig. 2). Highest binding responses with the best resolution between the different AgNPs concentrations were observed on the hMT1A-coated FC. IgG showed threefold lower dose-dependent binding activity towards the AgNPs than the hMT1A. Almost no binding was observed on the blank channel and only very low binding was observed on the FC coated with OVA. High binding capability of hMT1A towards AgNPs agrees with the known biological function of this protein. As an alternative to MT, bacterial metal-resistance proteins might be also suitable for detection of AgNPs [27]. Utilization of human MT in the sensor offers an additional value due to its relevant activity, which enables screening for AgNPs which could be biologically active in humans.

### hMT1A-sensor sensitivity evaluation

**Fig. 3:**
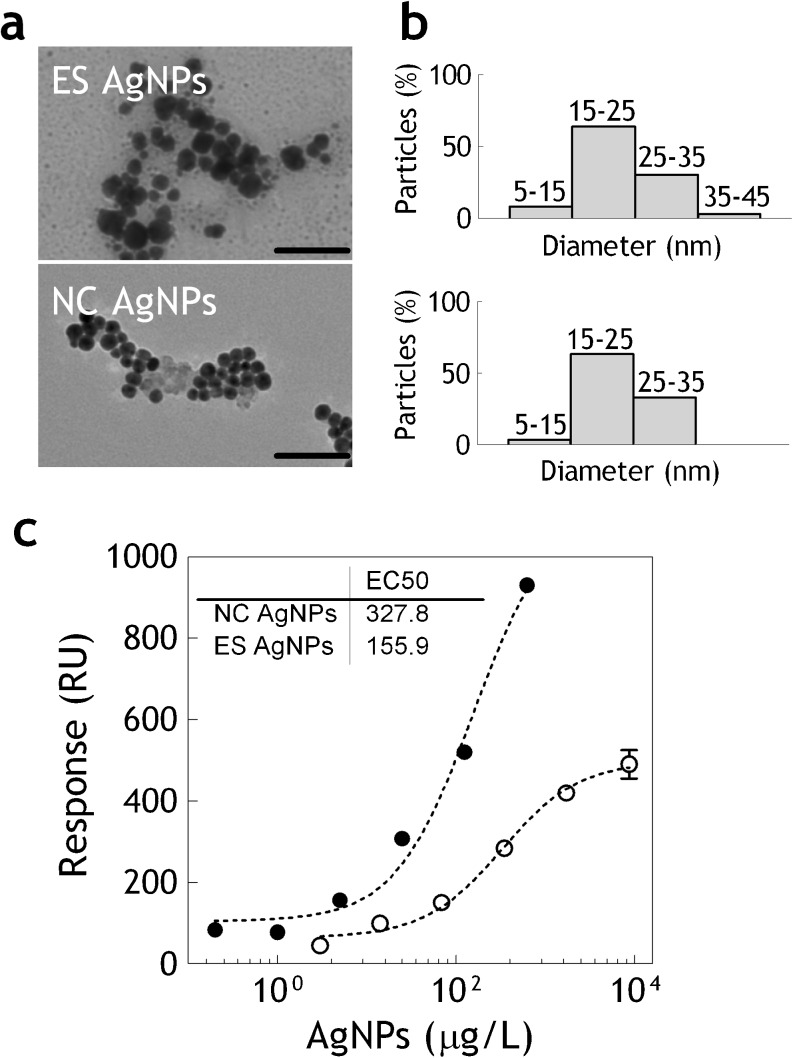
**a** Transmission electron microscopy (TEM) images of the ES AgNPs (*upper image*) and 20 nm NC AgNPs (*lower image*). **b** Size distribution of the ES AgNPs (*upper graph*) and NC AgNPs (*lower graph*) obtained by TEM image analysis. **c** Dose response curves measured on hMT1A-coated flow channel with ES AgNPs (*filled circles*) and with NC AgNPs (*open circles*). *Error bars* show standard deviations between three independent experiments. *Dotted lines* stand for the 4P fitted sigmoidal curve and the inset shows EC_50_ values derived from it

**Fig. 4:**
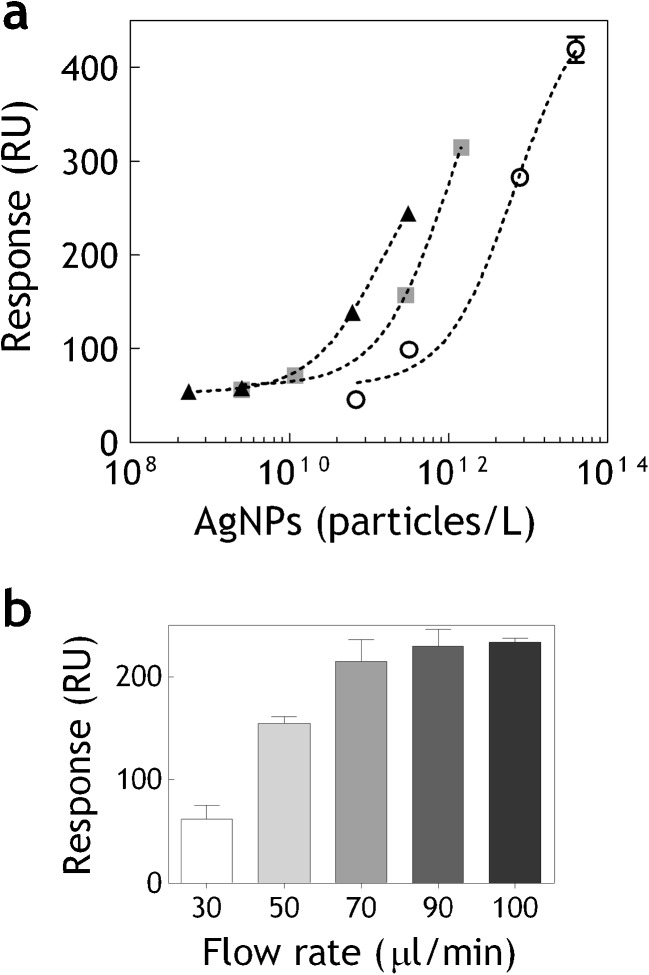
**a** Dose response curves measured on hMT1A-coated flow channel with different sizes of BioPure NanoComposix AgNPs—20 nm (*open circles*), 60 nm (*gray squares*), 100 nm (*black triangles*). *Error bars* show standard deviations between three independent experiments. **b** Maximal binding responses (RU) measured on hMT1A coated flow channel with 110 nm BioPure NanoComposix AgNPs at different flow rates (in microliters per minute)

AgNPs differ in size, morphology, and surface coating. When introduced into the food and the environment, both their physical and chemical properties might change. Also, the way the AgNPs were initially synthesized greatly influences their properties. In most toxicological studies commercially available AgNPs have been used with limited characterization of the suspensions, and sometimes with unspecified capping agents and little knowledge of potential impurities or stability [32]. The contamination levels, and the form of the AgNPs occurrence in the food products is also yet to be determined [9]. Therefore, in this study, two different types of AgNPs were used for sensor characterization: electrochemically synthesized polydisperse (ES) AgNPs and NC AgNPs stabilized by citrate. The NC AgNPs were used to characterize the interaction of the hMT1A with pure monodisperse AgNPs, whereas ES AgNPs were used as a model for the nanosilver which is more likely to appear in food and environmental samples. The ES AgNPs are attractive for industrial applications because they can be produced in a cost-effective manner and require no capping agent for stabilization [6, 8, 29]. For both types of AgNPs size, zeta potential and concentration were determined by means of TEM, Zetasizer and sp-ICPMS respectively. ES AgNPs exhibited a polydisperse suspension with 70 % of the NPs having a diameter around 20 nm with a low zeta potential (−37 mV), containing also some residual silver ions and small ionic clusters, see Electronic Supplementary Material Fig. S1. The NC AgNPs exhibited monodisperse suspension with a higher zeta potential (−18 mV). The hMT1A-sensor showed dose-dependent responses with both types of AgNPs (Fig. 3) with twice higher sensitivity towards ES AgNPs (EC_50_ = 156 μg/L) in comparison to the NC AgNPs (EC_50_ = 328 μg/L). The differences in sensor’s sensitivity towards these two types of AgNPs can be attributed both to citrate capping of the NC AgNPs and to the presence of ionic silver and small ionic clusters in the ES AgNPs, which have better diffusion and accessibility to the binding domains of the hMT1A. To elucidate the effect of the AgNPs size on sensor’s performance 20, 60, and 110 nm NC AgNPs were injected at increasing concentrations into the hMT1A-sensor. The comparison, based on the number of particles bound, showed that the sensitivity of the sensor increases with the AgNPs size (Fig. 4a). Increasing the flow rate only affected the responses until 70 μl/min, which was chosen as the working flow rate in all the experiments. Higher flow rates did not produce higher signals with 100 nm AgNPs (Fig. 4b). The higher responses of the larger AgNPs most probably result from the enhancement of the SPR signal which is proportional to the mass of the binding analyte and also from the coupling of localized surface plasmons of the AgNPs to the surface plasmon polariton of the gold surface. This SPR signal enhancement effect by metal NPs, mediated by their dielectric function, has been commonly employed for signal enhancement in several sensors [33, 34]. The detection range of the sensor described in this study for AgNPs is comparable to the MT-based electrochemical sensors previously developed for silver ions detection [22, 23]. Currently, the World Health Organization guidelines for drinking water quality indicate 100 μg/L as the safe upper limit for silver ions [35]. If the contamination levels of AgNPs in food will be established in the same range as for silver ions in drinking water, the sensitivity of the hMT1A-sensor described here should be adequate for screening purposes.

### hMT1A-sensor selectivity evaluation

**Fig. 5:**
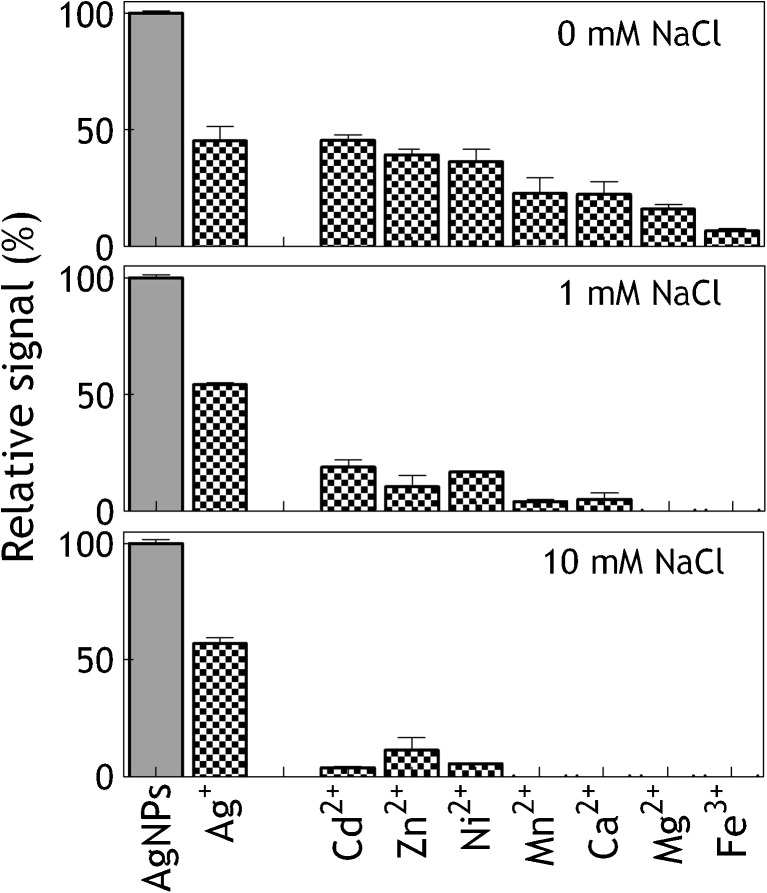
Cross-reactivity of hMTA1 with different ions at a concentration of 100 μg/L in comparison to the signal generated by 100 μg/L of ES AgNPs at different salt concentrations (0, 1 and 10 mM NaCl). *Error bars* show standard deviations between three independent experiments

Detection of AgNPs in food and environmental samples requires assessment of hMT1A-sensor selectivity towards other possibly reactive sample matrix components as well the selectivity towards the silver ions. Therefore, Cd^2+^, Zn^2+^, Ni^2+^, Mn^2+^, Ca^2+^, Mg^2+^, Fe^3+^, and Ag^+^ were introduced to the hMT1A-sensor at a concentration of 100 μg/L and their responses were compared to the responses obtained with the 100 μg/L of ES AgNPs (Fig. 5). Under low salt conditions (0 mM NaCl), hMT1A showed about 40 % cross-reactivity with Cd, Zn, Ni ions, about 20 % cross-reactivity with Mn, Ca, Mg ions and under 10 % cross-reactivity with Fe ions. Binding of the metal ions to hMT1A is in agreement with hMT1A’s biological activity and has been previously utilized in several biosensors for metal ions detection. C.M. Wu et al. have shown binding of Cd^2+^, Zn^2+^, Ni^2+^ but not of Mn^2+^, Ca^2+^, and Mg^2+^ to rabbit MTII using SPR [28]. By increasing the salt concentration (1 mM NaCl), the cross-reactivity of most of the ions was significantly reduced roughly to 15 %. At 10 mM, NaCl the cross-reactivity of Mn, Ca, Mg, and Fe ions was not detectable and the cross-reactivity of Cd, Zn, and Ni ions was reduced further to 4, 10, and 5 %, respectively. In comparison to ES AgNPs, silver ions generated 50 % lower responses at the same mass percentage, without dependence on the salt concentration. The responses of the ES AgNPs were not affected by the salt concentration neither, see Electronic Supplementary Material Fig. S2. These results indicate that’s the major interference with the signal generated by the AgNPs on the hMT1A-sensor should be expected from the silver ions, possibly present in the sample. Most likely, the food or water samples contaminated with AgNPs will also contain silver ions, due to both impurity of the added/migrated AgNPs and partial dissolution of the AgNPs. Thus, the ability to detect both forms of silver, ionic, and in nanoparticle state is beneficial for screening purposes. In case the final application of hMT1A-sensor will require selective screening for AgNPs alone, silver ions can be removed from the sample by means of dialysis or centrifugal filtration.

### Measurements in food and environmental matrices

**Fig. 6:**
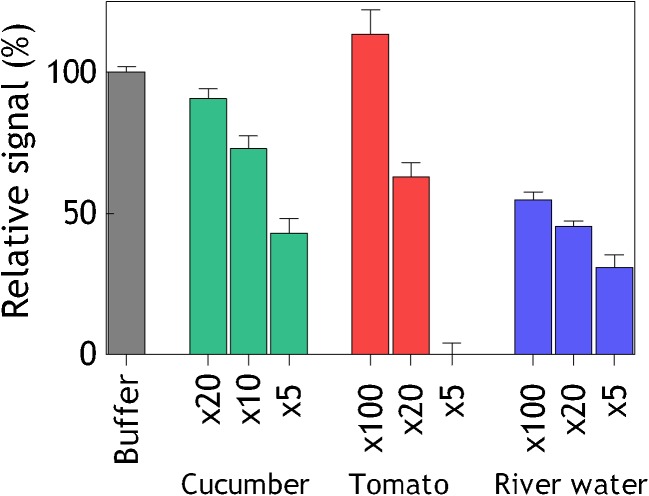
Sample matrix effect on the hMT1A-sensor performance. 100 μg/L of electrochemically synthesized AgNPs were spiked into diluted cucumber (*green bars*), tomato (*red bars*), and river (*blue bars*) water extracts and injected to the hMT1A-coated flow channel. The relative signal of the spiked samples was calculated as a percentage of the response obtained with buffer solution containing the same amount of AgNPs (*gray bar*). *x* stands for dilution fold of the sample extract. *Error bars* show standard deviations between three independent experiments

To evaluate the ability of the hMT1A-sensor to detect AgNPs in food and water samples, fresh cucumber, tomato and river water extracts were prepared at different dilutions (from 5- to 100-fold) and spiked with 100 μg/L ES AgNPs. The responses generated by the spiked samples were compared to the response generated by 100 μg/L ES AgNPs in buffer (Fig. 6). The observed matrix effect on the assay was lower in the samples with higher dilution factor and varied between different matrices. In the fresh cucumber extract, already a dilution of 20-fold generated almost 100 % response. In tomato, the sample had to be diluted 100 fold in order to achieve the same responses as in buffer. In river water at 100-fold dilution only 60 % of the response in buffer was obtained. The origin of the matrix effects observed here are evidently different in each sample. Not only can the sample matrix components interfere with the binding of the AgNPs to hMT1A, but they can also affect properties of the AgNPs. pH, ionic strength and composition, temperature, and nanoparticle concentration all interact to affect aggregation or stabilization of AgNPs. For example, in freshwater systems, organic matter and sulfide, with a high silver affinity, dominate the speciation and determine the AgNPs bioavailability [32]. Similar mechanisms are expected to affect the AgNPs physical and chemical properties in food, depending on the product formulation. There is not much known about the AgNPs fate in different food matrices, mainly due to the lack of reliable analytical methods for detection, quantification and characterization of AgNPs in complex matrices, and also due to the lack of sufficient AgNPs-containing product inventory. The hMT1A-sensor, described here was shown to detect AgNPs at the microgram-per-liter level in spiked fresh vegetables and river water samples. Only a minimal sample preparation was needed to be able to detect the AgNPs presence in the sample within 10 min.

## Conclusions

In this study, we have shown for the first time, that AgNPs can be directly detected in their intact form using hMT1A protein in combination with SPR-based sensor. hMT1A offers the advantage of detection bioavailable AgNPs and was proved to be a favorable protein ligand for capturing AgNPs on the sensor chip surface. The hMT1A-sensor showed sensitivity in the microgram-per-liter range, displaying the highest sensitivity towards larger and uncoated AgNPs. The cross-reactivity towards ions was reduced by increasing sodium chloride concentrations, but not eliminated for ionic form of silver, suggesting the need in a selective sample-prep for nanoform confirmation. The potential application possibilities of this sensor were demonstrated by successfully detecting AgNPs in fresh vegetables and river water extracts. The spiked samples were identified within 10 min, without the need in complex sample preparation steps. The sensor’s chip showed high robustness and stability over a period of several months. The utilization of the sensor described here for routine detection of AgNPs in food and environmental samples, will provide a rapid and automated screening method needed for efficient environmental and food safety monitoring.

## Electronic supplementary material

Below is the link to the electronic supplementary material.

ESM 1 (PDF 165 kb)
