# Supplementary material for: Development of surface plasmon resonance-based sensor for detection of silver nanoparticles in food and the environment
Source: Anal Bioanal Chem. 2012 Mar 27;403(10):2843–50. doi: 10.1007/s00216-012-5920-z (PMC3380250; doi:10.1007/s00216-012-5920-z)

Analytical and Bioanalytical Chemistry

Electronic Supplementary Material

Development of surface plasmon resonance-based sensor for detection of silver nanoparticles in food and the environment

Sabina Rebe Raz, Maria Leontaridou, Maria G.E.G. Bremer, Ruud Peters, Stefan Weigel

Figure 1: Single particle ICPMS picture of ES AgNPs at a concentration of 14 ng/L.

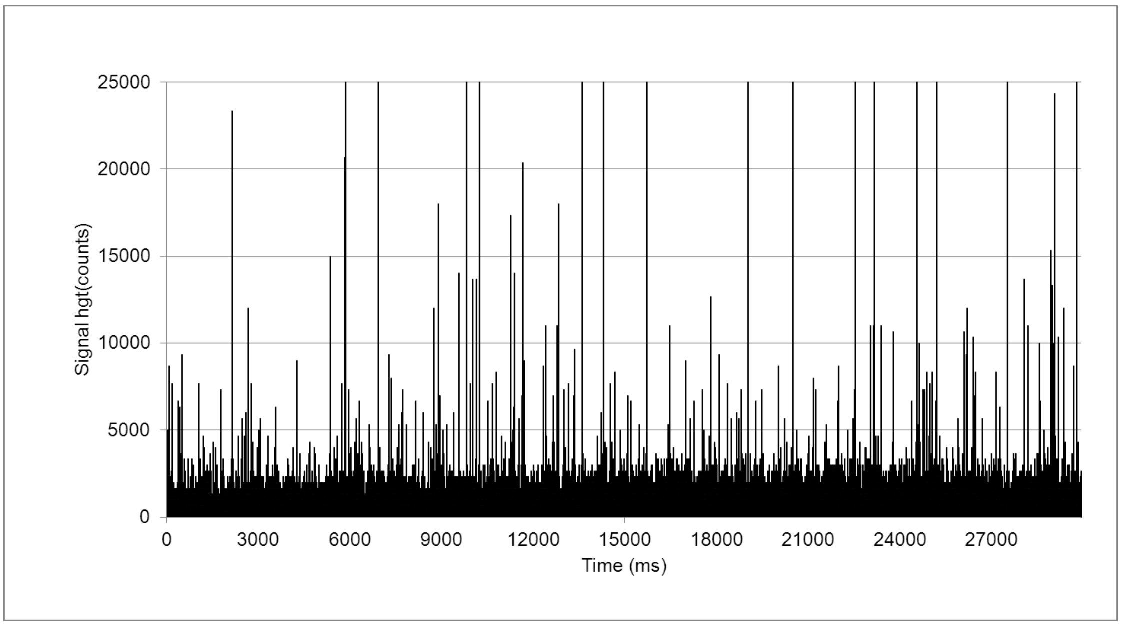

Figure 2: NaCl concentration effect on the responses produced by ES AgNPs.

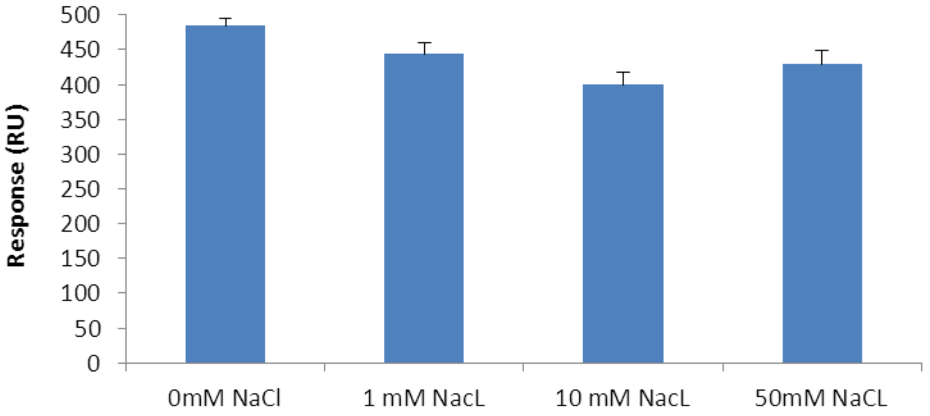

Supplement: Supplementary file 1 — (PDF 165 kb) [file 216_2012_5920_MOESM1_ESM.pdf]
